# An integrated genomic and biochemical approach to investigate the potentiality of heirloom tomatoes: Breeding resources for food quality and sustainable agriculture

**DOI:** 10.3389/fpls.2022.1031776

**Published:** 2023-01-04

**Authors:** Pasquale Tripodi, Antonietta D’Alessandro, Gianluca Francese

**Affiliations:** Council for Agricultural Research and Economics (CREA) Research Centre for Vegetable and Ornamental Crops, Pontecagnano, Italy

**Keywords:** tomato heirlooms, quality, carotenoids, vitamin C, SNP array, population structure, genotype × year interaction, breeding

## Abstract

A revival of interest in traditional varieties reflects the change in consumer preferences and the greater awareness of the quality of locally grown products. As ancient cultivars, heirlooms have been selected for decades in specific habitats and represent nowadays potential germplasm sources to consider for breeding high-quality products and cultivation in sustainable agriculture. In this study, 60 heirloom tomato (*Solanum lycopersicum* L.) accessions, including diverse varietal types (beefsteak, globe, oxheart, plum, and cherry), were profiled over two seasons for the main chemical and biochemical fruit traits. A medium–high level of heritability was found for all traits ranging from 0.52 for soluble solids to 0.99 for fruit weight. The average content of ascorbic acid was ~31 mg 100 g^−1^ of fw in both seasons, while the greatest variability was found for carotenoids with peaks of 245.65 μg g^−1^ of fw for total lycopene and 32.29 μg g^−1^ of fw for β-carotene. Dissection of genotypic (G) and seasonal (Y) factors highlighted genotype as the main source of variation for all traits. No significant effect of Y and G × Y was found for ascorbic acid and fruit weight, respectively, whereas a high influence of Y was found on the variation of lycopene. Molecular fingerprinting was performed using the 10K SolCAP array, yielding a total of 7,591 SNPs. Population structure, phylogenetic relationships, and principal components analysis highlighted a differentiation of plum and cherry genotypes with respect to the beefsteak and globe types. These results were confirmed by multivariate analysis of phenotypic traits, shedding light on how breeding and selection focused on fruit characteristics have influenced the genetic and phenotypic makeup of heirlooms. Marker–trait association showed 11 significantly associated loci for β-carotene and fruit weight. For β-carotene, a single variant on chromosome 8 was found at 12 kb to *CCD8*, a cleavage dioxygenase playing a key role in the biosynthesis of apocarotenoids. For fruit weight, a single association was located at less than 3 Mbp from *SLSUN31* and *fw11.3*, two candidates involved in the increasing of fruit mass. These results highlight the potentiality of heirlooms for genetic improvement and candidate gene identification.

## 1 Introduction

The roadmap designed by FAO to address trends and challenges of the next few decades highlights the triple burden of malnutrition consisting of food scarcity, nutrient deficiencies, and obesity. These factors often coexist in both developing and industrialized countries, with consequences for public health and costs for society (FAO, 2017). Changes in dietary patterns and the rediscovery of higher-quality foods are essential to reverse these trends. The concerns of consumers for a varied diet ensuring a healthy lifestyle and the increased awareness of the safety of the environment and biodiversity posed great attention to traditional food and locally grown crop varieties (Sproesser et al., 2019; Petrescu et al., 2020). Among these, heirlooms are traditional cultivars generally not used at a large agricultural scale, being routinely cultivated by local farmers as open-pollinated varieties and preserved mostly at a small community level. These cultivars are recognized for their uniqueness related to taste and flavor properties, as well as for the value given by local communities as a cultural heritage of a particular territory (Dwivedi et al., 2019; Esposito et al., 2020). In addition, they enclose a broad variability in terms of shape, color, and several other traits underlying the overall quality. The genetic makeup of tomato (*Solanum lycopersicum*) heirlooms is the sum of a complex history beginning from the first phases of domestication that occurred in Central-South America in the Andean and Mexico regions till its introduction in Europe and its diffusion in the rest of the globe in the 16th century after the discovery of the New World (Razifard et al., 2020). Both domestication and post-domestication processes led to a series of changes shaping the diversity of tomatoes through the development of highly differentiated morphotypes (Paran and van der Knaap, 2007). Nowadays, cultivated tomato is one of the most important vegetables in the food economy, ranking among the first 12 crops with a production of 187 million tons on a harvested area of over 5 million hectares (FAOSTAT, 2021). Global production mostly reflects modern hybrid cultivars that, although more productive and uniform, are often described with a poor organoleptic and nutritional value compared with heirlooms (Wang and Seymour, 2017). Indeed, industrial agriculture has been primarily focused on the cultivation of a limited number of cultivars able to meet commercial criteria (e.g., uniformity, yield) rather than consumer preferences (Cheema and Dhaliwal, 2005). Therefore, local germplasm represents a potential source of useful alleles to improve the adaptation, productivity, and quality of crops (van de Wouw et al., 2010). The importance of heirlooms is increasing over the past few years as a benchmark for sustainable agriculture and for their use in organic farming. Being adapted to natural and/or marginal environments, heirlooms could perform better than modern-breed cultivars under minimal input conditions (Brouwer et al., 2016; Karaca and Ince, 2019). On the contrary, most hybrids and/or modern varieties have been bred for intensive farming resulting in a loss of allelic combinations useful for resilient agriculture (Tian et al., 2021). In this context, the development of long-established varieties in small-scale farms and in rotation cropping systems could improve the sustainability of cultivation, further strengthening regional economies (Brouwer et al., 2016).

Tomatoes are well-known for being rich in health-beneficial compounds, being a major source of carotenoids and providing an essential amount of vitamin C. Lycopene (non-provitamin A) is the most abundant carotenoid present in red fruits, of which tomato is the primary font among vegetables (Li et al., 2021). A dietary intake of food products containing lycopene exerts several health benefits by preventing the incidence of several chronic diseases including breast and prostate cancer as well as coronary infarction (Arballo et al., 2021). β-Carotene (provitamin A) is a major pigment precursor of the synthesis of retinol affecting cell differentiation and sight and exerts a scavenging activity against free radicals, thus exhibiting anticancer and antioxidant properties (Sowmya Shree et al., 2017). Finally, ascorbic acid (vitamin C) is a water-soluble antioxidant molecule with multiple functions as a cofactor of several enzymes (Padayatty and Levine, 2016), and in the genetic and epigenetic regulation of genes, it plays a central role in the defense response against biotic and abiotic stresses in plants (Venkatesh and Park, 2014). Ascorbic acid cannot be synthesized by humans, and its deficiency could lead to serious illness (Naidu, 2003); therefore, adequate consumption of ascorbic acid through diet is required. Beyond favorable health-related substances, the quality of tomatoes is given by several compounds defining the overall soluble solids content and the acidity, both underlying the taste and flavour of fruits (Tigist et al., 2013).

Quality improvement is among the major targets to achieve in tomato breeding programs following the increase of the standards required in marketplaces over the past years (Capobianco-Uriarte et al., 2021). Progress in metabolomics and genomics provides powerful tools to investigate germplasm resources and unlock their hidden potential, thus laying the foundation for next-generation breeding strategies (Razzaq et al., 2022).

The release of the whole genome sequence of tomato and the advancements in next-generation sequencing technologies facilitated the development of resources for high-throughput genomic scan (Nguyen et al., 2019). A single nucleotide polymorphism (SNP) array was built as a platform for high-density genotyping of tomato collections (Sim et al., 2012a). The array developed from transcriptome sequences of diverse accessions belonging to *S. lycopersicum*, *S. lycopersicum* var. *cerasiforme*, and *S. pimpinellifolium* L. was designed using 10,000 probes leading to a total of 8,784 SNPs of which 7,720 are of high quality, thus being appropriate for genomic scale analysis of germplasm collections representing different market classes (Sim et al., 2012b). The advantages of the array include the possibility of multiplexing, the increase of rapidity and throughput of the genome scan, and the effectiveness in allele calling with high call rates (Thomson, 2014). Furthermore, with the position of SNPs fixed, the genotyping information can be easily compared and/or implemented across multiple collections. So far, the array has been used to investigate germplasm collection (Blanca et al., 2012), interspecific crossing population (Chetelat et al., 2019), mapping of loci (Brog et al., 2019), and genome-wide association studies (Sauvage et al., 2014).

In the present study, 60 heirloom tomato accessions were evaluated across two cultivation seasons with the aims to a) determine the variable level of main carotenoids, ascorbic acid, and chemical traits and estimate the genotypic and environmental effects underlying the variation of traits; b) investigate the population structure and phylogenetic relationships using the available 10K SNP array in order to corroborate metabolic and genomic profiles of the collection; and c) implement an early marker–trait association to identify potential candidates underlying the quality-related traits. The results of the study will be of interest for the enhancement of heirloom tomato varieties by promoting their use in local markets and sustainable agriculture and as a source of valuable quality-related traits for genetic improvement programs.

## 2 Materials and methods

### 2.1 Plant material and experimental trials

The plant material consisted of 60 cultivated tomato (*S. lycopersicum*) accessions, retrieved from the collection available at the genebank of the Research Centre for Vegetable and Ornamental Crops (CREA, Pontecagnano, SA, Italy) or provided by farmers’ associations. The genotypes’ panel was composed of diverse heirloom varietal types including beefsteak (11), cherry (16), globe (8), oxheart (3), and plum (22) (**Table 1**) and included a wide phenotypic diversity in terms of fruit morphology, shape, and color (**Supplementary Table 1**). The accessions studied represent the most important heirlooms cultivated in Italy or in international farmers’ markets. Prior to the experiments, to increase the number of seeds, accessions were subjected to a cycle of controlled self-fertilization under glasshouse conditions at CREA-OF.

**Table 1:** Plant material used in this study.

Typology	Name
Beefsteak	Beefsteak (BF), Black Tula (BL), Canestrino (CA), Costoluto Fiorentino (CF), Costoluto Genovese (CG), Gigante RR (GR), Marmande (MA), Pantano (PN), Pera Abbruzzese (PA), Pomodoro di Sorrento (PS), Snow White (SW)
Cherry	Black cherry (BY), Black truffle (BT), Flat cherry (FC), Frassino locale (FN), Giallo a grappoli (GG), Giallo pro (GP), Gold cherry (GC), Kiros (KI), Nero Pro (NP), Piennolo (PI), Pomodoro del Vesuvio (PV), Principe borghese (PB), Regina chiaro (RC), Small red (SR), Tondino nocerese (TN), Vesuviano (VE)
Globe	Ailsa Craig (AC), Blue P20 (BE), Brione black (BB), Caro Rich (CR), Geneva (GE), Mei fan orange (MF), Moneymaker (MM), Rutgers (RT)
Oxheart	Belmonte calabro (BM), Big Pear (BP), Cuor di bue rosso (CB)
Plum	Austin’s Red Pear (AR), Bottle red pear (BR), Cento Scocche (CS), Chulu Mani (CM), Corbarino (CO), Fiaschello (FL), Fiascone (FS), Itallong (IT), Mezzo Fiasco (MS), Nocera (NO), Orange Pear (OP), Ovale (OV), Pizzuto (PZ), Pomodoro scatolone (PT), Rio Grande (RG), Roma long (RL), San Marzano (SM), San Marzano lungo (SL), SMEC (SC), Yellow cherry (YC), Yellow ovate (YO), Yellow round (YR)

For the 60 heirloom accessions, the cultivar category is indicated according to fruit type, common name, and acronym (in brackets). Additional details are in **Supplementary Table 1**.

Experimental trials were performed across two seasons during 2019 (Y1) (max T° = 28.00°C, min T° = 15.12°C, UR% = 71.94, precipitation = 5.00 mm) and 2020 (Y2) (max T° = 28.33°C, min T° = 15.02°C, UR% = 72.85, precipitation = 5.38 mm) in the experimental farm of CREA (40°39′N, 14°52′E) following a randomized block design with three replicates and five plants/replicates. Plants were grown in single rows adopting distances of 1.50 m between the rows and 0.5 m apart on the rows. Black mulch was used to cover the rows. Cultivation was managed according to standard agronomic practices: plants were irrigated throughout the entire cultivation period using a drip irrigation system, and fertilization was provided through irrigation water. The transplant occurred in May and harvesting in August. At the maturity stage, a bulk of 10 fruits from each accession/replicate, for a total of 30 fruits/accession, was harvested, weighted (fruit weight, fw), and subjected to chemical analysis (soluble solids, acidity, pH). For the biochemical analysis, three replicates consisting of eight fruits for each accession were collected, pooled frozen in liquid nitrogen, and then stored at −80°C until analysis.

### 2.2 Chemical traits

The chemical traits were as follows: titratable acidity (TA) and pH (pH) were measured on a 10% (w/v) aqueous tomato extract and NaOH 0.1 M as a titrating reagent using a pH-Matic 23 analyzer titroprocessor equipped with a pH electrode including a temperature sensor (model 5011T) (Crison Instruments, Barcelona, Spain) and soluble solids content (SSC) was measured on 0.5 ml of a tomato liquid extract using a digital refractometer (HI 96801, Hanna Instruments, Padua, Italy; Refracto 30PX, Mettler-Toledo, Novate Milanese, Italy). TA was expressed as g citric acid/L juice, and SSC was expressed as Brix degree.

### 2.3 UPLC ascorbic acid determination

Ascorbic acid (AsA) was determined using an Ultimate 3000 UPLC system (Thermo Fisher Scientific, Sunnyvale, CA, USA). Aqueous extracts (1 g of fruit plus 3 ml of 6% metaphosphoric acid in distilled water) were homogenized for 30 s using an Ultra-Turrax (IKA, Wilmington, NC, USA) and then centrifuged at 1,975×*g* for 15 min. The extraction was repeated twice on the pellet; the supernatant was collected each time and finally made to 10 ml with the extracting solvent. The extracts were then filtered using 0.2-μm polytetrafluoroethylene (PTFE) filters. A final volume of 5 μl of the sample was injected on a Kinetex (75 × 4.6 mm, 100 Å, particle size 2.6 mm) column (Phenomenex, Torrance, CA, USA). The mobile phase constituted 0.02 mol L^–1^ of H_3_PO_4_ aqueous solution at a flow rate of 0.35 ml min^–1^. Quantification of AsA was performed at 254 nm using a calibration curve of an authentic chemical standard of ascorbic acid from Sigma-Aldrich (St. Louis, MO, USA). The AsA content was adjusted as mg 100 g^−1^ of fw (fresh weight).

### 2.4 RP-HPLC carotenoids detection

β-Carotene, *trans*-lycopene, and *cis*-(9, 13, 15) lycopene were analyzed by reversed-phase high-performance liquid chromatography (RP-HPLC) using a Waters E-Alliance HPLC system constituted by a separations module with a quaternary pump, an autosampler, and a photodiode array detector (Waters, Milford, MA, USA); data were acquired and analyzed with Empower software (Waters). Five grams of fruit pericarp was used. A reversed-phase analytical polymeric C30 column (250 × 4.6 mm i.d.; 3 μm particle diameter; YMC, Wilmington, NC, USA) was used for chromatographic separations. The separation parameters were a flow rate of 0.8 ml min^−1^ and 0.005 AUFS (absorbance units full scale). Solvents used for sample preparation and extractions were of analytical grade, while those for HPLC analysis (methyl-t-butyl ether, methanol, ethyl acetate, and tetrahydrofuran) were of HPLC grade; all were obtained from Merck (Darmstadt, Germany). *Trans*-carotenoid standards (lycopene and β-carotene) used in the HPLC analyses were purchased from Sigma Chemical Co. (Sigma-Aldrich Company, St. Louis, MO, USA). Results were expressed as μg g^−1^ of fw.

### 2.5 Molecular genotyping

DNA was isolated by collecting six young leaves (100 mg) from each accession. Two leaves were considered for each accession/replicate. Once harvested, the tissue was lyophilized in microcentrifuge 2-ml tubes and then disrupted to a fine powder using a Tissue Lyser II (Qiagen, Hilden, Germany) at 30 strokes per second for 30 s. Nucleic acid isolation was then performed using the PureLink™ Genomic Plant DNA Purification Kit (Thermo Fisher Scientific, Waltham, MA, USA). DNA concentration and quality were measured by absorbance at 260 and 280 nm, respectively, using both UV–Vis spectrophotometer (ND-1000; NanoDrop, Thermo Scientific, Wilmington, DE, USA) and Qubit 2.0 Fluorometer based on Qubit dsDNA HS Assay (Thermo Fisher Scientific, Waltham, MA, USA). The DNA solution was then diluted to a working concentration with distilled water. Genotyping was performed using the Illumina 10K SolCAP Tomato Infinium array (Illumina Inc., San Diego, CA, USA) yielding a total of 7,720 SNPs (Sim et al., 2012a). SNP calls, through the translation of the signals of fluorescent dyes into genotype groups, were obtained using GenomeStudio version 1.7.4 (Illumina Inc., San Diego, CA, USA).

### 2.6 Statistical analysis

All traits were subjected to analysis of variance (ANOVA) using the generalized linear model (GLM) implemented in IBM SPSS Statistics, version 25.0 (IBM Corp., Armonk, NY, USA) to determine the overall differences within the studied accessions. A two-factorial linear model was used to determine the effects of genotype (G), year (Y), and their interaction (G × Y) on trait performance. Mean square values (MS) were used to estimate the magnitude of the observed effect, while the total sum of squares in percentage (TSS%) was calculated by dividing the TSS of the effect by the total TSS. Means were compared using Tukey’s honestly significant difference test (*P*< 0.05). The coefficient of variation (CV) as a percentage was expressed as the ratio of the standard deviation to the mean value multiplied by 100. Broad-sense heritability (*H*^2^) was estimated according to the formula:


$$H^{2}=\;\frac{\sigma_{G}^{2}}{\sigma_{G}^{2}+\sigma_{y}^{2}+\epsilon }$$

where *σ*^2^_G_ is the genetic variance, *σ*^2^*_y_* is the variance due to season, and *ε* is the error.

Correlations across the genotypes for phenotypic traits were calculated using the Pearson test at *P*<0.05. The correlogram was constructed and visualized using the *psych* and *corrplot* R packages implemented in R version 3.0.2 (R Core Development Team, 2013). Principal component analysis (PCA) for phenotypic traits was inferred using the computer package XLSTAT 2012.1.

### 2.7 Genomic diversity and phylogenetic analysis

SNP markers with more than 10% of missing genotypes were removed. The resulting SNP matrix consisting of 7,591 SNPs was used for downstream analysis. Population structure was determined using the parametric Bayesian model-based clustering method implemented in STRUCTURE v.2.4 (Pritchard et al., 2005). The admixture model analysis and the Markov chain Monte Carlo (MCMC) method for allele frequency estimation and identification of the best number of population (*K*) were used. Runs were done using 50,000 burn-in cycles followed by 100,000 MCMC iterations, with the number of subpopulations (*K*) ranging between 1 and 10 with five independent runs for each *K*. The most probable numbers of subpopulations were determined according to Evanno’s method using Structure Harvester (Earl and vonHoldt, 2012). Accessions were considered to belong to a specific subpopulation if its membership coefficient (qi) was ≥0.50, whereas the genotypes with qi lower than 0.5 at each assigned *K* were considered as admixed. A neighbor-joining phylogenetic tree was built using the maximum composite likelihood method with 1,000 bootstraps. Analyses were conducted in MEGA X software (Kumar et al., 2018). Principal component analysis was conducted in R by the function *prcomp* (package stats), and the biplot was drawn using the ggplot2 R package (Wickham, 2016).

### 2.8 Marker–trait association

Association analysis was performed using four models: i) the simple regression model (GLM, naive model), ii) the general linear model incorporating the population structure Q matrix (GLM-Q), iii) the general linear model incorporating principal components as a covariate (GLM-PCA), and iv) the mixed linear model (MLM) incorporating the population structure covariates (*Q*) and the kinship (*K* matrix) estimated using the identity by state (IBS) for accounting relationships among individuals. Phenotypic data from the two independent experiments were implemented. The significance threshold for marker–trait association was determined after Bonferroni multiple test correction with genome-wide *α* = 0.05. All analyses were performed in Tassel v 5.2.82 (Bradbury et al., 2007). To confirm associations, the MLM and the compressed mixed linear model (CMLM) implemented in the GAPIT R package (Wang and Zhang, 2021) were performed. Manhattan and quantile–quantile (*Q*–*Q*) plots for GWAS results were produced using the R package *CMplot*. Significant association signals were checked for their physical position on the *S. lycopersicum* SL4.0 genome. Underlying genes and their functions were determined considering the tomato ITAG release 4.1.

## 3 Results

### 3.1 Analysis of variance

A wide phenotypic diversity was observed in the germplasm collection studied. The ANOVA results highlighted significant differences (*P* > 0.001) among the heirloom tomato varieties for all traits when pooled data from the two seasons were analyzed (**Table 2**). Fruit weight showed the greatest *R*^2^ and *F* values, whereas the lowest values for both parameters were found in pH and SSC. On average, we found a high heritability, with values above 70% for all the essayed traits except for soluble solids, pH, and *trans*-lycopene. Ascorbic acid content ranged from 57.49 to 15.11 mg 100 g^−1^ of fw. Although the range of variation was smaller during the second year, the average content was similar in both seasons being ~31 mg 100 g^−1^ of fw. Total lycopene and β-carotene showed average values of 86.14 and 2.50 μg g^−1^ of fw, respectively, with ranges across the 2 years of 245.65–0.00 μg g^−1^ of fw for total lycopene and 32.29–0.02 μg g^−1^ of fw for β-carotene, thus suggesting a high level of variation among accessions for carotenoids. This was confirmed by the coefficient of variation which reached peaks of 109.67% and 155.47% in β-carotene, during Y1 and Y2, respectively, and values above 60% for lycopene. Only chemical parameters and ascorbic acid exhibited a CV below 26%.

**Table 2:** Descriptive statistics, broad-sense heritability (*H*^2^), range, mean, and coefficient of variation in percentage (in bracket) for the traits analyzed on 60 heirloom accessions during two seasons (Y1 and Y2).

Trait^a,b^	Acronym	Mean square	*R*^2^	*F* ratio	Prob > *F*^c^		*H*^2^		Range (Y1)	Mean (CV)		Range (Y2)	Mean (CV)
Fruit weight	FW	88,613.55	0.99	798.58	*		0.98		333.65 - 6.78	84.57 (98.18)		357.00 - 7.25	90.49 (98.18)
Brix	SSC	8.72	0.52	11.97	*		0.58		9.86 - 5.59	7.33 (12.66)		9.33 - 4.93	6.27 (15.27)
pH	pH	0.19	0.51	11.69	*		0.62		5.05 - 4.17	4.49 (3.75)		4.85 - 4.08	4.39 (2.76)
Acidity	AC	0.13	0.81	21.27	*		0.73		0.86 - 0.30	0.51 (24.24)		0.68 - 0.23	0.45 (21.06)
Ascorbic acid	AsA	425.20	0.53	12.69	*		0.72		57.49 - 15.11	30.76 (25.04)		40.18 - 20.45	30.91 (14.01)
β-Carotene	βcar	130.02	0.55	48.44	*		0.89		18.59 - 0.02	2.30 (109.67)		32.29 - 0.13	2.70 (155.47)
*Trans-*lycopene	TransLyc	30,373.57	0.63	13.40	*		0.66		147.26 - 0.38	61.33 (63.24)		245.65 - 0.00	104.29 (69.55)
*Cis-*lycopene	CisLyc	51.41	0.63	19.38	*		0.78		9.94 - 0.00	3.59 (69.29)		8.93 - 0.00	3.06 (72.96)
9 *Cis*-lycopene	CisLyc9	12.51	0.65	20.40	*		0.78		3.92 - 0.00	1.36 (73.85)		4.77 - 0.00	1.63 (78.52)
13 *Cis*-lycopene	CisLyc13	13.88	0.55	13.44	*		0.70		6.31 - 0.00	2.07 (70.97)		4.05 - 0.00	1.30 (80.13)
15 *Cis*-lycopene	CisLyc15	0.14	0.53	12.71	*		0.76		0.70 - 0.00	0.17 (90.84)		0.60 - 0.00	0.13 (94.74)

^a^FW is expressed in grams; SSC as Brix degree; AsA in mg 100 g−1 of fw; carotenoids (β-carotene and lycopene) as μg g−1 of fw.

^b^For FW, SSC, pH, and AC, a total of 30 fruits were used to determine each variable mean for Y1 and Y2; for AsA, βcar, TransLyc, CisLyc, CisLyc9, CisLyc13, and CisLyc15, a total 8 fruits were used to determine each variable mean for both Y1 and Y2.

^c^Significance at *P < 0.001.

The results of the combined analysis of variance for fruit weight and chemical and biochemical traits across the two seasons are given in **Table 3**. The main source of variation was due to G, which accounted for on average 64.04% of the total variation, expressed by TSS%, ranging from 98.62% for fruit weight to 51.13% for pH. A high level of significance (*P*< 0.001) was found for all the traits. Partitioning of TSS% in the other components affecting the variation highlighted a generally smaller influence of Y (average 4.76%) and G × Y (average 16.57%) compared with G on the analyzed parameters. Fruit weight showed the strongest effect of genotype, being the only trait exhibiting no significant effect for G × Y. Ascorbic acid was instead slightly influenced by seasonal factors, thus showing no significance for the Y effect. Among bioactive compounds, β-carotene had the highest genotypic effect (81.24%), whereas a different degree of variability was found for the rest. *Trans*-lycopene showed a higher seasonal effect than *cis*-lycopene. For the latter, *cis*-9 lycopene was more strongly affected by genotype than the other two isomers.

**Table 3:** *F* values and significant levels for genotype (G), season (Y), and G × Y effects for the 10 traits on 60 tomato heirloom genotypes over 2 years.

	*df*	Fruit weight		Soluble solids		pH		Acidity	
		TSS%	*F*	TSS%	F	TSS%	*F*	TSS%	*F*
Genotype (G)	59	98.62	872.32*	51.68	31.95*	51.13	22.66*	65.49	36.95*
Year (Y)	1	0.12	62.09*	20.29	740.18*	7.72	201.92*	5.89	195.98*
G × Y	59	0.11	0.99^ns^	11.57	7.15*	18.19	8.06*	10.59	5.97*
Error	600	1.15		16.45		22.95		18.02	
		Ascorbic acid		β-Carotene		*trans*-lycopene		*cis*-lycopene	
Genotype (G)	59	53.15	27.34*	81.24	86.04*	54.50	32.50*	63.40	39.77*
Year (Y)	1	0.01	0.33^ns^	0.30	18.77*	10.10	355.36*	1.08	40.05*
G × Y	59	27.08	13.93*	8.85	9.37*	18.34	10.9*	19.30	12.10*
Error	600	19.76		9.60		17.06		16.21	
		9 *cis*-lycopene		13 *cis*-lycopene		15 *cis*-lycopene			
Genotype (G)	59	64.60	38.53*	54.59	34.23*	53.35	37.47*		
Year (Y)	1	1.18	41.53*	7.19	265.92*	1.48	61.40*		
G × Y	59	17.18	10.25*	22.01	13.81*	30.69	21.56*		
Error	600	17.05		16.21		14.48			

ns, not significant.

*Indicates significance at p ≤ 0.001.

### 3.2 Fruit weight and chemical analysis

**Figure 1:**
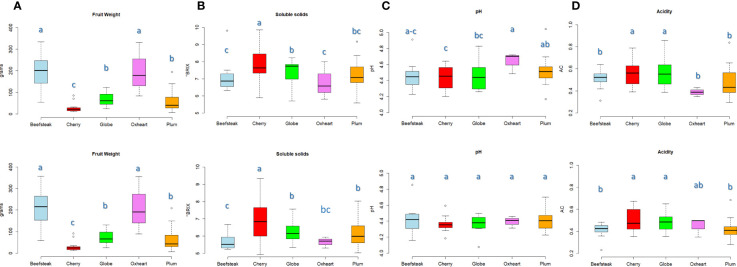
Variation for fruit weight and chemical traits in the five heirloom groups across two seasons. Boxplots showing the average value and quartiles (first and third) for traits scored in the first (above) and the second (below) seasons, respectively. The measurement scale for each trait is reported on the *Y*-axis. Means with different letters are significantly different (*P* = 0.05) according to Tukey’s.

Marked divergences were found among the considered varietal types for fruit weight. Beefsteak and oxheart types showed the highest average fruit weight (**Figure 1A**), being 200.33 and 197.90 g during the first season, respectively, with an increment of about 10% during the second season. Cherry, plum, and globe types showed a lower fruit weight with average values not exceeding 80 g in both seasons. ‘Belmonte Calabro’ and ‘Costoluto Genovese’ showed the highest average fruit weight with values exceeding 300 g in both seasons (**Supplementary Table 2**). On the contrary, ‘Bottle Red Pear’ exhibited the lowest fruit weight with values below 10 g in both years.

Soluble solids content was on average higher in cherry types with values of 7.92 and 6. 92 °Brix during the first and the second seasons, respectively. Oxheart types, instead, exhibited the lowest values (**Figure 1B**). The highest contents were reached with 9. 86 °Brix (‘Small red,’ cherry) during Y1 and 9.33 °Brix (‘Nero Pro,’ cherry) during Y2. We observed a general decrease of soluble solids during the second trial in all accessions, with a few exceptions including ‘Nero Pro,’ ‘Black cherry,’ and ‘Gold cherry’ (cherry), ‘Itallong’ (plum), and ‘Geneva’ (globe) (**Supplementary Table 2**). Less variation was found among cultivar groups for pH and acidity (**Figures 1C, D****)**. All accessions exhibited a pH<5, being on average 4.49 and 4.39 during Y1 and Y2, respectively. Only ‘San Marzano lungo’ (plum) showed values of 5.05 during the first season (**Figure 1C**). Oxheart types showed the highest average pH during Y1, being significantly different from the other cultivars, whereas no significant variation was found in all groups of cultivars in the second season. Cherry and globe types exhibited a higher level of acidity than the other groups (**Figure 1D**) with values above 0.55 and 0.48 g citric acid/L during the first and the second seasons, respectively. Acidity values higher than 0.65 g citric acid/L were found in ‘Rutgers’ (globe), ‘Chulu Mani’ (plum), and ‘Flat cherry’ (cherry) in both trials (**Supplementary Table 2**).

### 3.3 Vitamin C and carotenoids

**Figure 2:**
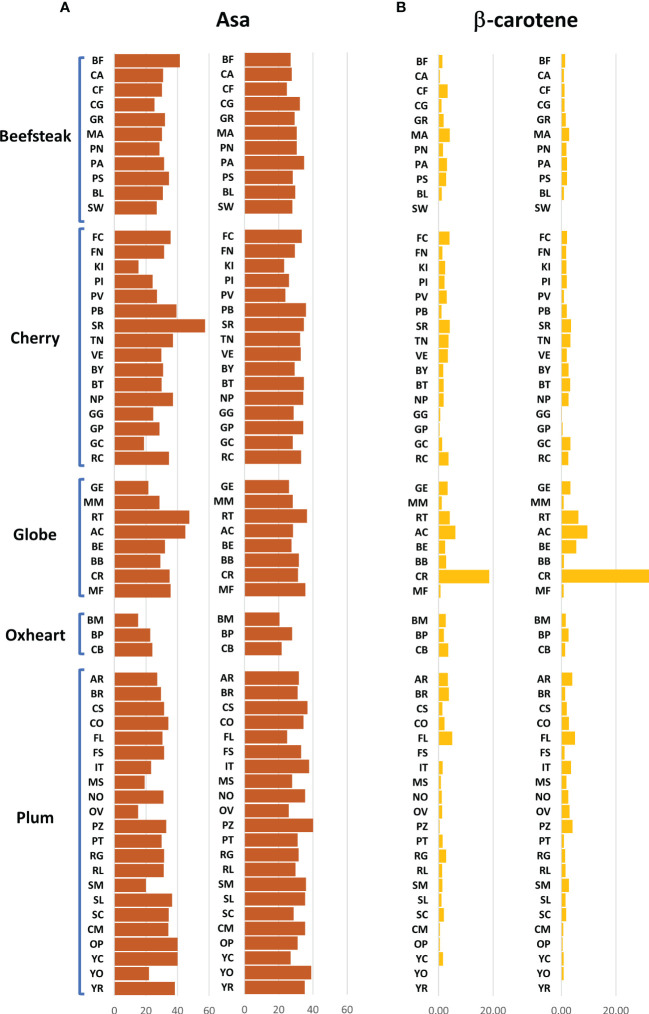
Variation for ascorbic acid and β-carotene in 60 heirloom genotypes in the two years of evaluation. Vertical bars show the average values for each accession. For each trait, data of the first and the second seasons are on the left and the right, respectively. Genotypes are ordered based on cultivar group. Acronyms are listed in **Supplementary Table 1**.

**Figure 3:**
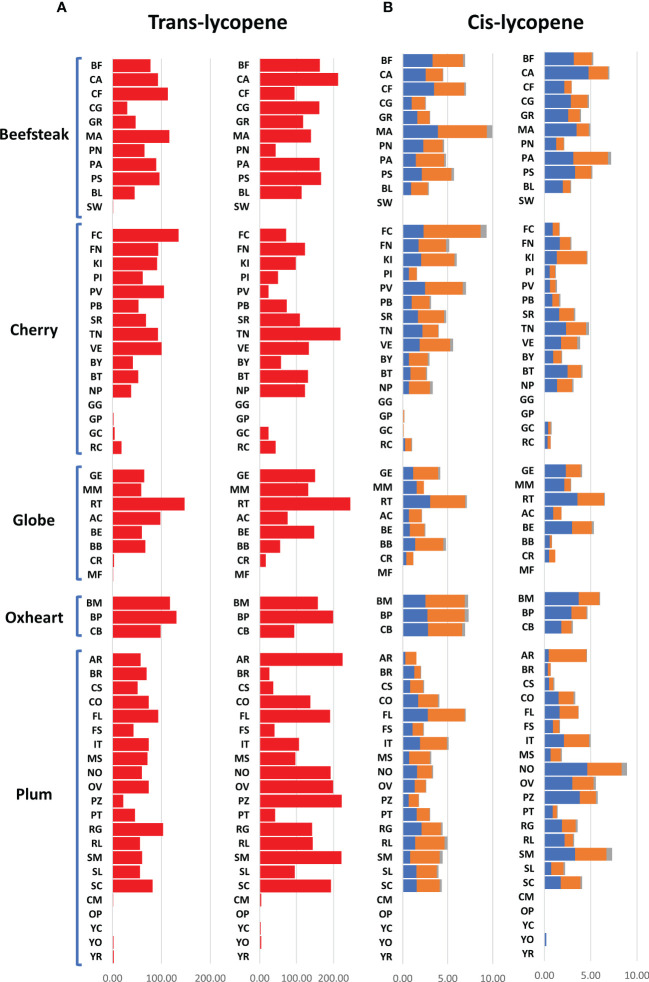
Variation for *trans*- and *cis*-lycopene in 60 heirloom genotypes in the 2 years of evaluation. Vertical bars show the average values for each accession. For each trait, data of the first and the second seasons are on the left and the right, respectively. 9-*Cis*, 13-*cis*, and 15-*cis* lycopene are indicated in blue, orange, and gray colors, respectively. Genotypes are ordered based on cultivar group. Acronyms are listed in **Supplementary Table 1**.

The analytical results for ascorbic acid, β-carotene, and lycopene content showed a high variability between accessions (**Figures 2**, **3**). The average content of AsA was similar across trials, being 36.00 and 36.17 mg 100 g^−1^ of fw in the first and second seasons, respectively. A higher coefficient of variation was found in the first trial (CV = 25.04%) than in the second one (CV = 14.01%). A different trend was instead observed within each category: plum and oxheart types showed average higher AsA levels during the second season; contrariwise, beefsteak, cherry, and globe types showed lower levels (**Figure 2A**). Outstanding levels of AsA were found for Small red (57.49 mg 100 g^−1^ of fw) during the first season (**Figure 2A**; **Supplementary Table 2**) although the trend was not confirmed in the subsequent year. The oxheart type ‘Belmonte Calabro’ exhibited the lowest AsA content with values of 15.16 and 20.45 mg 100 g^−1^ of fw during Y1 and Y2, respectively. With regard to β-carotene, globe types had the greatest levels in both seasons. Only a few accessions showed levels above 5 μg g^−1^ of fw, including the globe heirlooms ‘Ailsa craig’ and ‘Rutgers’ and the plum ‘Fiaschello.’ In addition, ‘Caro Rich’ an orange cultivar specifically selected for the high β-carotene content showed outstanding levels of this compound with peaks of 32.29 μg g^−1^ of fw (**Figure 2B**, **Supplementary Table 2**). Considering the single lycopene forms essayed, *trans*-lycopene was preponderant, representing over 95% of total lycopene in all accessions. On average, a higher content of *trans*-lycopene was found in the second year, with a more marked difference (percentage of variation >100%) in plum types (**Figure 3A**, **Supplementary Table 2**). A different trend was found for *cis*-lycopene, with a higher content during the first season in all cultivar groups except for plum types **(** **Figure 3B**). 9-*Cis*- and 13-*cis* were the prevalent isomers, both reaching values not exceeding 4.8 μg g^−1^ of fw, whereas the maximum content of 15-*cis* was 0.60 μg g^−1^ of fw. As expected, in all cultivar groups, yellow accessions showed the lowest levels of lycopene. Among these, the cherry ‘Regina chiaro’ had a considerable level of both *trans*- and *cis*-lycopene being 19.38 and 43.89 μg g^−1^ of fw during the first and second years, respectively.

### 3.4 Multivariate analysis and correlation between traits

**Figure 4:**
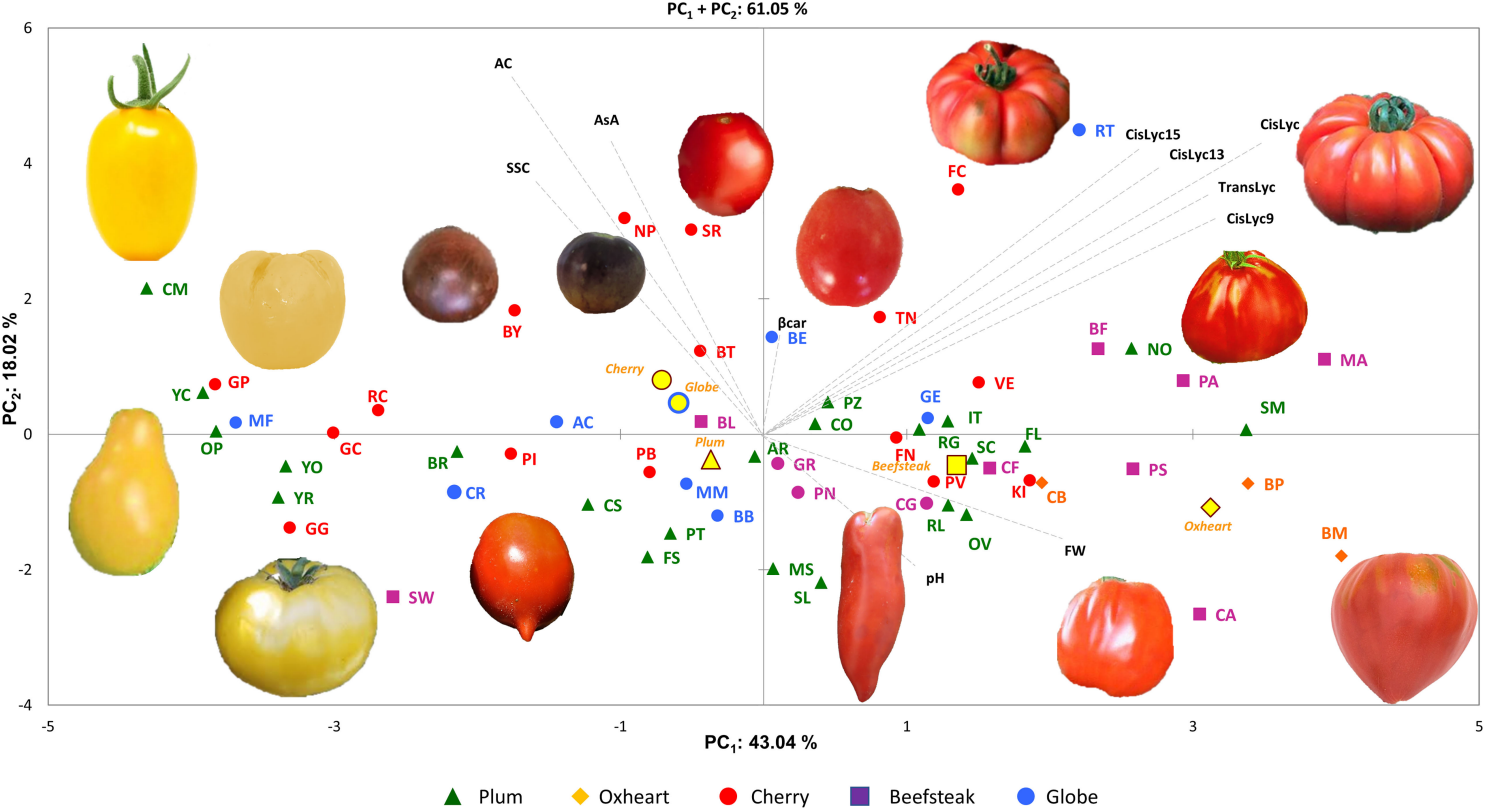
Principal component analysis. Loading plot of the first (PC_1_) and second (PC_2_) principal components showing the variation for 10 traits scored across two seasons. Accessions of different cultivar groups are represented by different colored symbols. Examples of the morphological characteristics of the fruits for the studied accessions are included in the figure. Color and symbols are listed in the graph’s legend. The first and second component centroids for each cultivar group are indicated by filled yellow symbols with shape and edge color according to cultivar groups (see legend). The direction and distance from the center of the biplot indicate how each trait scored contributes to the first two components. Trait acronyms are listed in **Table 1**.

**Figure 5:**
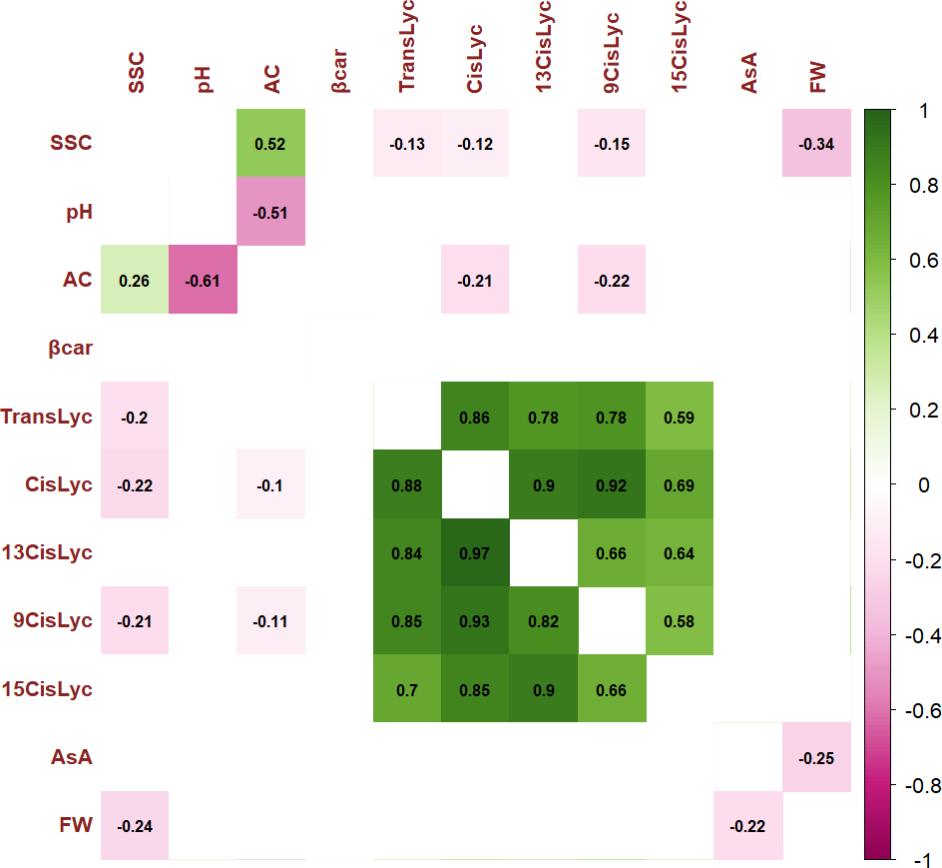
Pearson’s rank correlation coefficients between pairs of phenotypes. Correlation coefficients are indicated in each cell. Colored correlations are those with a *P*-value<0.05. Color intensity is directly proportional to the coefficients. On the right side of the correlogram, the legend color shows the correlation coefficients and the corresponding colors. The correlogram for traits scored during Y1 is placed below the diagonal, and the correlogram for traits scored during Y2 is placed above the diagonal. Trait acronyms are listed in **Table 1**.

For the 11 analyzed traits, the PCA in the first two dimensions revealed 61.05% of the total variance (**Figure 4**). Cherry, plum, and beefsteak accessions were evenly distributed in both the negative and positive axes of the graph. Oxheart types were distributed only on the positive section of the first axis (PC_1_) and the negative of the second one (PC_2_), respectively, whereas globe types were distributed on both PC_1_ and PC_2_ excluding both the positive and negative sections of the first and second axes. The first component, which explained 43.04% of the total variance, was positively correlated with carotenoids, fruit weight, and pH and negatively correlated with ascorbic acid, acidity, and soluble solids. The second component, which explained 18.02% of the total variance, was positively correlated to all traits except pH and fruit weight. *Cis*-lycopene and acidity were the main factors discriminating the genotypes under study accounting for 17.32% and 33.15% of the total variation of the first and second components, respectively. The projection of the accessions on the two-dimensional PCA graph highlighted the differentiation of cherry and globe types which were mostly distributed in the negative axis of the first component, plum accessions which were mostly placed in the negative axis of the second component, and oxheart and beefsteak cultivars which were mainly distributed in the positive loadings of the first component. Based on PCA, it was possible to discriminate the cultivar groups according to specific features: cherry cultivars showing small fruits and a higher content of soluble solids and vitamin C and beefsteak and oxheart exhibiting greater fruit weight of the collection. The Pearson correlation matrix (*P*< 0.05) for the two growing seasons is reported in **Figure 5**. Correlations mainly occurred within the same trait categories. In both seasons, fruit weight was negatively correlated with soluble solids and ascorbic acid, *trans*- and *cis*-lycopene were negatively correlated with soluble solids, and *cis*-lycopene was negatively correlated with acidity, whereas a significant positive correlation was found between acidity and soluble solids. All correlations among the different trait categories showed a coefficient<0.4.

### 3.5 Genomic diversity

Genomic diversity analysis was inferred using 7,591 biallelic SNPs present in over 90% of individuals (**Data Sheet 1**). The identified SNPs were located in all 12 chromosomes, with an average density of one SNP every 99.31 kilobases, and 54.73% of SNPs resulted to be positioned at a distance lower than 20 kilobases. The highest number of SNPs was found on chromosome 11 (*n* = 1,059) and the lowest on chromosome 12 (*n* = 383) (**Supplementary Figure 1**). The biggest gap of 7.36 megabases was found on chromosome 1 at 26.85 megabase position. Nucleotide substitution included transitions (A→G and T→C) and transversions (C↔G, A→C, T↔G, A↔T), and we found a higher frequency of transitions (69.61%) than transversions (30.39%). On average, the level of heterozygosity of the collection was 2.82%. Fifty accessions showed a heterozygosity level below 2%. Only five genotypes exhibited a heterozygosity between 5% and 15%, namely, ‘Corbarino’ (6.24%), ‘Pomodoro di Sorrento’ (14.98%), ‘Ovale’ (11.08%), ‘Flat Cherry’ (11.68%), and ‘Beefsteak’ (7.91%). ‘Fiaschello’ and ‘Small red’ showed a high level of heterozygosity with 26.04% and 25.72%, respectively (**Supplementary Figure 2**).

**Figure 6:**
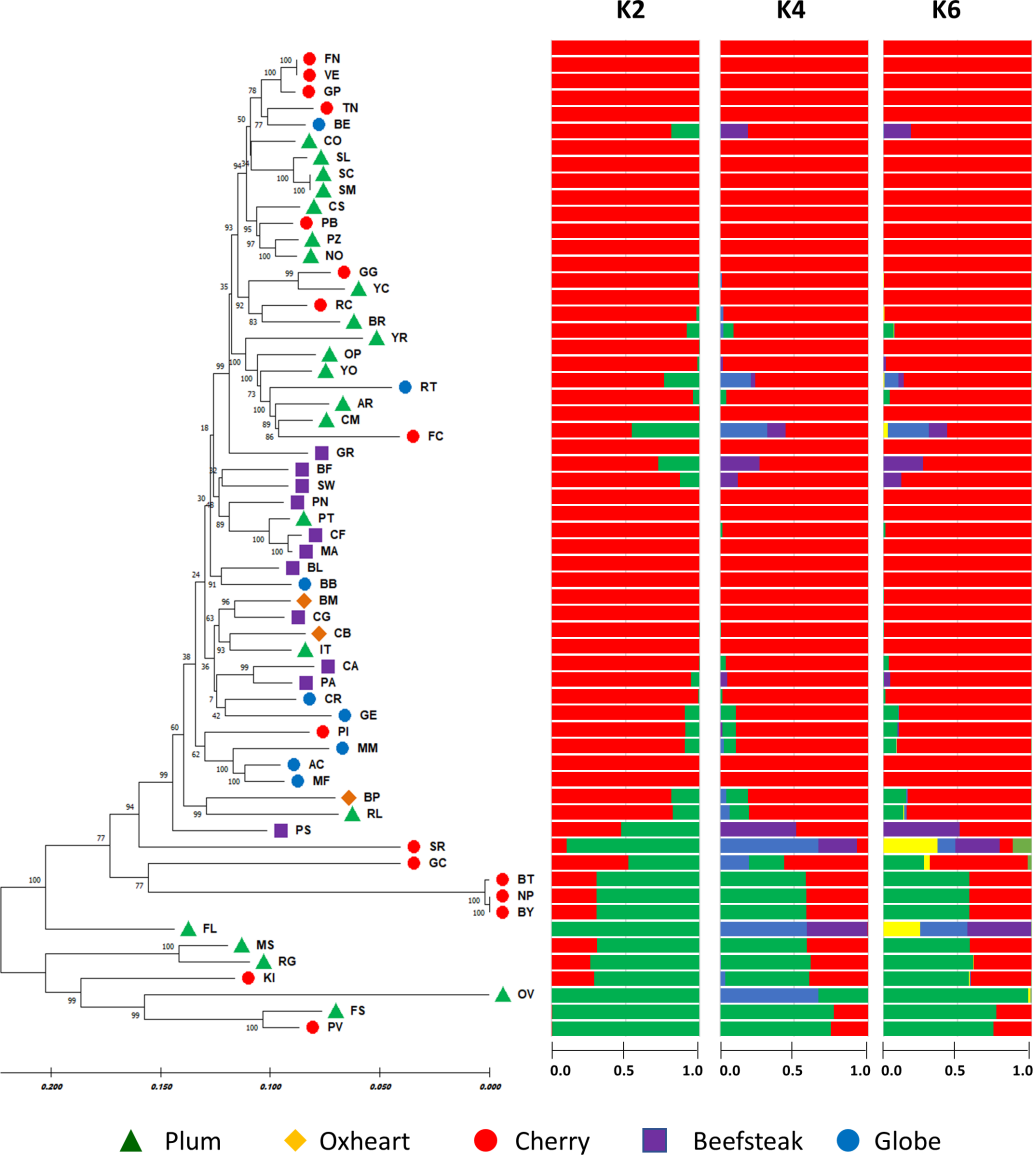
Phylogenetic analysis and population structure of 60 tomato accessions based on 7,591 SNPs. On the left, the neighbor-joining phylogenetic tree was computed using the maximum composite likelihood method. Numbers at the nodes are bootstrap values for 1,000 replicates. Accessions of different cultivar groups are represented by different colored symbols as listed in the legend. On the right, STRUCTURE analysis considering the most likely number of subpopulations *K* = 2 based on Evanno’s test. Clustering at *K* = 4 and *K* = 6 is shown. Horizontal solid bars for each genotype represent the allele frequency (indicated with numbers) for each *K*.

Based on STRUCTURE analysis (**Figure 6**), the collection was divided into *K* = 2, which was likely the number of subpopulations according to Evanno’s test (**Supplementary Figure 3**). The main group comprised 48 accessions including all beefsteak (excluding ‘Pomodoro di Sorrento’), globe, and oxheart genotypes plus cherry and plum individuals. The minor group included six cherry and six plum heirloom types. A high coefficient of membership (qi ≥ 0.9) was found for 40 accessions of cluster 1, whereas 3 accessions, namely, ‘Flat cherry,’ ‘Pomodoro di Sorrento,’ and ‘Gold cherry,’ showed a similar qi for the two *K* clusters, thus can be considered admixed. Within the second cluster, four accessions, viz. ‘Fiaschello,’ ‘Ovale,’ ‘Fiascone,’ and ‘Pomodoro del Vesuvio,’ had a very high cluster membership coefficient (qi) >0.99. The high coefficient membership for the accessions of cluster 1 was retained at *K* = 4 and *K* = 6, whereas a different degree of admixture was found for the accessions of cluster 2, particularly for ‘Small red,’ ‘Gold cherry,’ and ‘Fiaschello.’

The maximum likelihood-based phylogenetic tree using the neighbor-joining method provided a better resolution of the genetic diversity. The presence of two major clusters was confirmed, including 54 and 6 accessions, respectively. The dendrogram highlighted subgroups comprising the different varietal types studied: plum and cherry types tended to be separated from beefsteak, oxheart, and globe types. A higher level of similarity was found within plum and cherry types, particularly for cultivars ‘Frassino locale’ and ‘Vesuviano’ and ‘SMEC’ and ‘San Marzano’ and the black types ‘Nero Pro,’ ‘Black truffle,’ and ‘Black cherry.’

**Figure 7:**
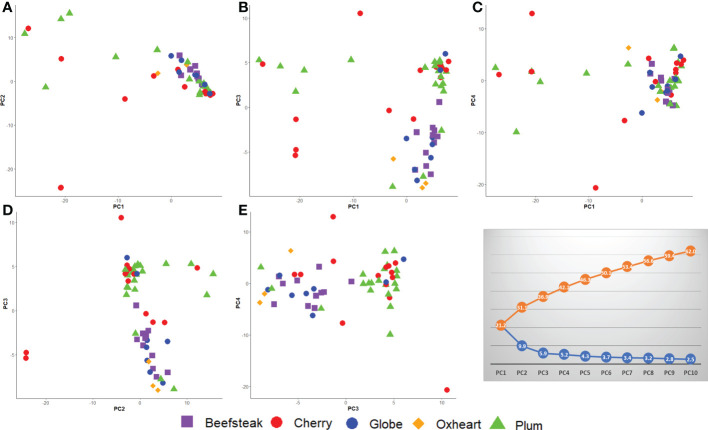
Loading plots in the first four components, showing the genomic diversity of 60 studied tomato accessions. **(A)** PC_1_ vs. PC_2_; **(B)** PC_1_ vs. PC_3_; **(C)** PC_1_ vs. PC_4_; **(D)** PC_2_ vs. PC_3_; **(E)** PC_3_ vs. PC_4_. The percentage of variation explained is shown in the grey plot. The orange line (above) indicates the cumulative variation of the components from 1 to 10; the blue line (below) indicates the variation explained by each component.

PCA confirmed the higher differentiation at the genomic level for plum and cherry heirlooms compared with the beefsteak and globe types. The distribution of accessions on PC_1_ vs. PC_2_, PC_1_ vs. PC_3_, and PC_1_ vs. PC_4_ (**Figures 7A–C**) highlighted the diversity of both plum and cherry types. The diversity of the collection was well resolved by PC_2_ vs. PC_3_ (**Figure 7D**) and PC_3_ vs. PC_4_ (**Figure 7E**), in which the two main clusters separating beefsteak, globe, and oxheart types from plum and cherry ones were shown.

### 3.6 Association analysis

**Figure 8:**
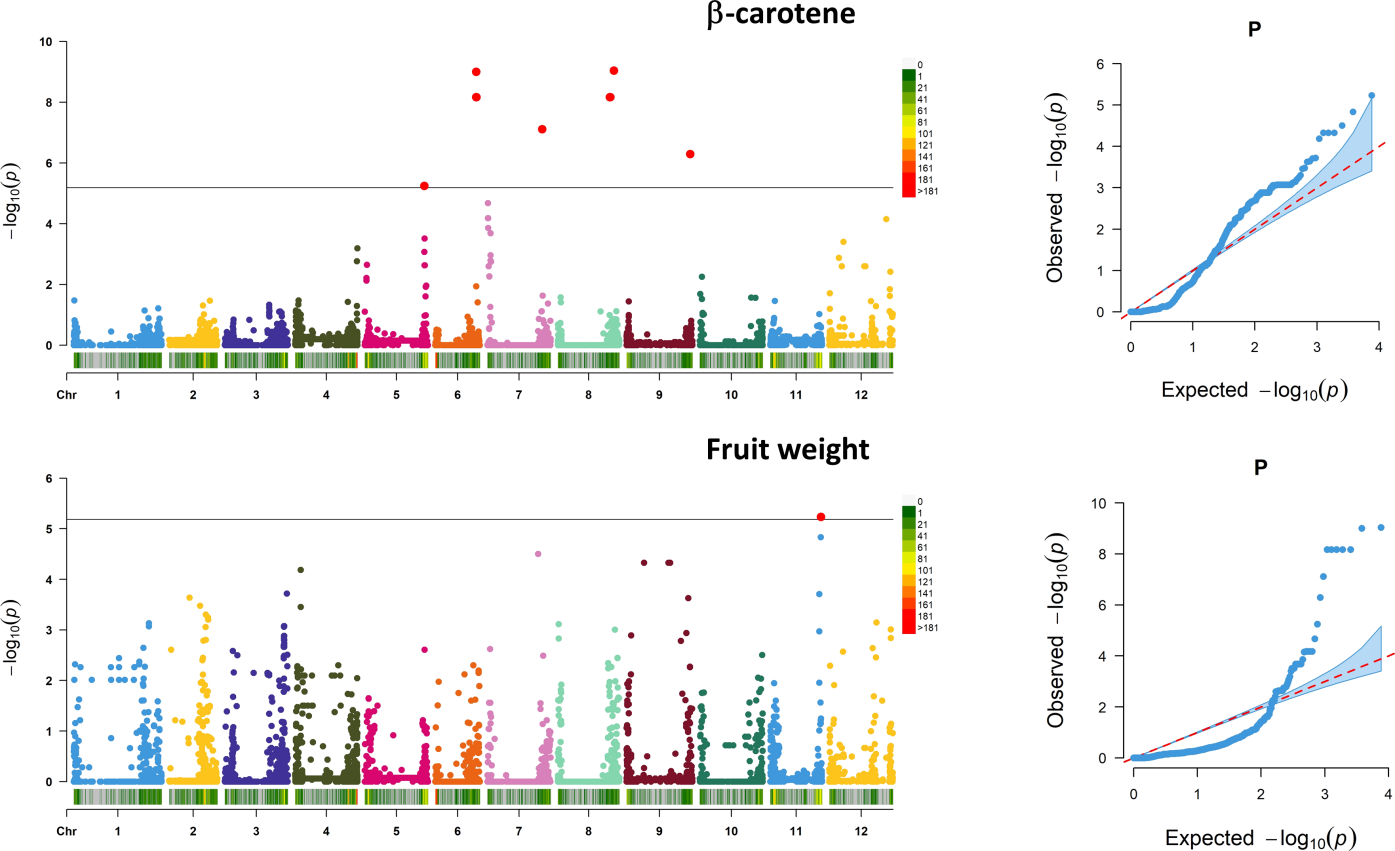
Marker–trait association analysis. Manhattan plots showing SNP associations for β-carotene (above) and fruit weight (below). Analysis has been performed considering 7,591 SNPs on 60 accessions evaluated across 2 years. The black horizontal line indicates a significant threshold (−log_10_ *P*-value) according to Bonferroni. The *X*-axis indicates the chromosome position and SNP density in 1 MB window following the legend on the right of each graph. For the associated traits, the QQ plot is reported on the left.

Associations between SNP alleles and traits were acquired based on different models. Significant associations were found in both GLM and MLM models only for fruit weight and β-carotene. The generalized linear models highlighted several spurious associations, as suggested by a comparison with probabilities predicted from a theoretical uniform distribution of *P*-values (**Supplementary Figure 4**). The GLM corrected for PCA components provided a better picture although only the MLM produced reliable associations. For the traits scored, a total of 11 significant marker associations were consistently identified in both seasons based on both GLM and MLM using 7,591 SNPs (**Table 4**). Associations were found on six chromosomes mostly in intergenic regions at a distance of 0.19 to 12.4 kb from the nearest genes. Ten out of the 11 signals were found for β-carotene, whereas only one SNP was found to be significantly associated with the variation of fruit weight. Manhattan plots and *Q*–*Q* plots showing the associations, their chromosomal positions, and Bonferroni threshold are in **Figure 8**. For β-carotene, the strongest signal [−log_10_(*P*) = 9.03] falling within a *pto-interacting protein 1-like* was found at the bottom of chromosome 8 (Snp_64706). A *Small nuclear ribonucleoprotein* located at the bottom of chromosome 6 [Snp_57377, −log_10_(*P*) = 9.00] was at 196 kb distance from the second peak detected. In the same region (**Table 4**), three additional associations were found: one within a kinase like *KIP1-like* protein [Snp_57374, −log_10_(*P*) = 8.17] and the remaining two [Bcyc_868, −log_10_(*P*) = 8.17; CL016102-0429_Snp_57352, −log_10_(*P*) = 8.17] at less than 1 Mb from a *CASP-like protein* and a *DUF674 domain-containing protein* . Another cluster on chromosome 8 [Snp_48550, −log_10_(*P*) = 8.16; Snp_18185, −log_10_(*P*) = 8.16] was found at 53-54 Mbp position at 12.40 kb distance from a *Carotenoid Cleavage Dioxygenase* 8 and at 1.90 kb distance from a *Cytochrome b-c1 complex subunit 7* . Another peak at the bottom of chromosome 7 [Snp_10686, −log_10_(*P*) = 7.10] was found at 3.29 kb from a *glycotransferase* , whereas the remaining associations at the bottom of chromosomes 9 [Snp_11670, −log_10_(*P*) = 6.29] and 5 [Snp_37209, −log_10_(*P*) = 5.24] were not associated to any known protein. For fruit weight, only the SNP SL1_00sc6004_2094360_Snp_44897 [−log_10_(*P*) = 5.23] was found in intergenic regions at 2.41 kb from a *Receptor-like serine/threonine-protein kinase NCRK* on chromosome 11. The peak was not significantly associated using the CMLM model implemented in GAPIT.

**Table 4:** Significant marker–trait associations detected traits using both general and mixed linear models.

Trait	SNP marker*	Chr	Position^2^	*F*	Marker *R*^2^	Major/minor allele	−log_10_ *P-*value	Candidate/nearby gene	SNP position relative to the candidate gene^b^	Candidate gene annotation
β-Carotene	Snp_64706	8	57.57	44.39	0.37	A/G	9.03	Solyc08g075330.3.1	0.0	pto-interacting protein 1-like
	Snp_57377	6	42.23	44.28	0.37	G/A	9.00	Solyc06g072280.3.1	+ 0.19	Small nuclear ribonucleoprotein E
	Snp_57374	6	42.23	22.21	0.37	C/A	8.17	Solyc06g072290.3.1	0.0	KIP1-like
	Bcyc_868	6	42.29	22.21	0.37	G/A	8.17	Solyc06g072350.3.1	− 0.75	CASP-like protein
	CL016102-0429_Snp_57352	6	42.34	22.21	0.37	A/G	8.17	Solyc06g072410.3.1	+ 0.94	DUF674 domain-containing protein
	Snp_48550	8	53.52	22.20	0.37	A/G	8.16	Solyc08g066650.3.1	− 12.40	Carotenoid cleavage dioxygenase 8
	Snp_18185	8	54.00	22.20	0.37	A/G	8.16	Solyc08g067020.5.1	+ 1.90	Cytochrome b-c1 complex subunit 7
	Snp_10686	7	56.55	32.86	0.27	G/A	7.10	Solyc07g043120.1.1	− 3.29	Glycosyltransferase
	Snp_11670	9	65.51	16.46	0.27	C/G	6.29	Solyc09g089650.1.1	+ 2.66	Hypothetical protein
	Snp_37209	5	61.18	13.42	0.22	C/A	5.24	Solyc05g051450.2.1	− 1.03	Unknown protein
Fruit weight	SL1_00sc6004_2094360_Snp_44897^c^	11	52.28	13.39	0.15	G/A	5.23	Solyc11g069590.2.1	+ 2.41	Receptor-like serine/threonine-protein kinase NCRK

For each trait, chromosomal position (in bp), main statistic and effects of each association, and corresponding annotated gene or close annotated gene are shown. F values, R2, and −log10 P-value refer to MLM.

*Snp = solcap_snp_sl.

^a^Distance expressed in million base pairs.

^b^Upstream and downstream SNPs close to candidate genes are specified with “–” and “+,” respectively. 0 indicates that SNPs fall within the candidate gene. The distance is expressed in kilobases.

^c^Not significantly associated using the CMLM model implemented in GAPIT.

## 4 Discussion

Intensive farming, urbanization, and marketing have impacted the diversity of agricultural systems, increasing the genetic erosion of crops. Globally, few plant species are dominating the agricultural scenario of which the production is based on a limited number of reference varieties (Martin et al., 2019). During the past 60 years, it has been estimated that the number of cultivars used in many crops decreased by over 70% posing a concrete risk to food security (Martin et al., 2019). Heirlooms, being evolved in their growth territory under typical agroclimatic conditions and following cultural preferences, are of paramount importance for preserving biodiversity. Beyond the recognized value as a reservoir of genes to confer resistance to various stresses, their history suggests an additional worth for promoting sustainable agricultural practices and providing a basis for high-quality food (Casañas et al., 2017). It is, therefore, crucial to investigate at the “omics” level the definition of strategies of conservation, utilization, and/or planning of new breeding programs. In this study, we characterized 60 heirloom genotypes for their genomic diversity and for the content of main compounds underlying quality across two independent experiments. The cultivar set studied enclosed a great assortment of morphotypes as confirmed by the weight of the fruits observed.

### 4.1 Biochemical characterization

All genotypes exhibited a wide level of variation for phytochemical traits, with the highest differentiation for the content of carotenoids. The estimation of heritability and of G × Y effect was performed to define how traits can be affected by environmental fluctuations, thus suggesting the use of the set of heirlooms across diversified conditions. A high degree of heritability is a sought-after goal in breeding, providing an estimate of response to selection for improving traits (Piepho and Möhring, 2007). The essayed traits showed a medium–high heritability which was maximized for fruit weight and β-carotene, highlighting how the long-term selection carried out by farmers has focused on the development of diverse cultivars in terms of morphology and colors. The effect of genotype was preponderant in all instances, confirming to be the main factor of variation for fruit weight. *Trans*-, *cis*-13 lycopene, and soluble solids content were instead majorly affected by seasonal factors. During the second season, a substantial increase of *trans*-lycopene up to +70% with respect to the previous one, as well as a decrease of soluble solids and *cis*-lycopene for 14.44% and 37.35%, respectively, was found. The accessions were grown in the same site, and only a slight variation in terms of temperature was found in the two seasons as well as an increase of 1% of humidity and 0.3 mm of rain/precipitation in the second year. It has been reported that water shortage could have impact on soluble solid (Zhang et al., 2017) and lycopene (Takács et al., 2020); however, these observations were retrieved from *ad-hoc* stressed field trials. Furthermore, the biosynthesis of lycopene is affected by sunlight, being inversely correlated with high irradiation levels (Brandt et al., 2006). Therefore, more investigations are required to define any effects of soil water on the variation of these compounds.

The ascorbic acid content varied among the accessions studied, independent of the fruit color at maturity, thus being similar in both red and yellow tomatoes. The average values were in accordance with other studies describing the content of vitamin C in vintage tomatoes (Panthee et al., 2013). Furthermore, the panel studied did not seem to provide an outstanding source of ascorbate, although the recommended daily dosage (60 mg/100 g) was ensured (Somanchi et al., 2017).

In agreement with Leiva-Brondo et al. (2012), we found the absence of significance of the environmental effect on the variation of AsA and a preponderant effect of G and G × E, thus confirming a key role of the genetic material on phenotypic values. Beyond genotype, the variable level of vitamin C in tomato is linked to several factors including the environment (Roselló et al., 2011), fertilization (Ye et al., 2020), and solar radiation (Dumas et al., 2003), which may have influenced the variation of the content found within certain accessions across the two trials.

Lycopene content instead varied according to fruit color when ripe. The highest amount was found in red fruited genotypes. This trend is expected since lycopene is the principal compound providing the red color to tomatoes, as well as the most abundant carotenoid. The lycopene concentration showed high variability between accessions. Previous attempts performed in different local cultivated varieties and wild tomato accessions reported average values in the range of ~35-60 mg/kg (Brandt et al., 2006; Adalid et al., 2010; Athinodorou et al., 2021) with peaks up to 167 mg/kg (Adalid et al., 2010). Despite in some of these studies lycopene has been estimated through spectrophotometry, the levels found can be considered comparable with HPLC as reported by Rao et al. (1998). These results agreed with our evaluation confirming how the collection essayed provides a good source of lycopene.

*Cis*-lycopene accounted for less than 10% of the total in all accessions except for Caro Rich (27.48%); furthermore, for many maturing yellow accessions, no trace of the *cis*-isomer was found. It has been reported that the *cis* form of lycopene is better adsorbed by the organism compared with the *trans* one due to the shorter length of the molecule, less tendency to aggregate, and higher solubility (Boileau et al., 2002). Fruit processing increases the bioavailability of lycopene through the isomerization of all-*trans* isomers to the *cis* form (Boileau et al., 2002; Unlu et al., 2007). In addition, the bioavailability of lycopene is also due to the content of β-carotene (Singh and Goyal, 2008), thus explaining the higher percentage of *cis*-lycopene found in Caro Rich. *Cis*-lycopene is subjected to heat-mediated isomerization, leading to the formation of 9-*cis*, 13-*cis*, or 15-*cis* isomers (Arballo et al., 2021). Among these, 9-*cis* exerts a higher antioxidant capacity than the other forms (Holzapfel et al., 2013).

The second main carotenoid responsible for the orange color in tomato is β-carotene, for which the average content ranges from 1 to 12 μg g^−1^ of fw (Marti et al., 2016). The values observed in the heirloom collection are in accordance with previous essays (Adalid et al., 2010; Panthee et al., 2013; Tripodi et al., 2021). Only Caro Rich provides an outstanding level of β-carotene, being a specific genotype improved for the content of provitamin A (Marti et al., 2016).

The correlation among traits confirmed the opposite trend between the content of soluble solids and the weight of fruits (Tripodi et al., 2022), suggesting, furthermore, a feasible way to select genotypes with improved vitamin C and lycopene by reducing fruit weight. In agreement with Labate et al. (2011), we did not find any correlation between lycopene and vitamin C, as well as lycopene and Brix, although, in the literature, positive correlations between the two latter traits are reported (Hyman et al., 2004) as the effect of the combination of ripening and accumulation of sugars.

Overall, the content of phytochemicals sheds light on the possibility to identify promising genotypes with enhanced nutritional quality to be used to promote breeding for high-quality products.

### 4.2 Genomic diversity

The collection evaluated in the present study was made up of half Italian germplasm and half by accessions of different origins, mostly retrieved from the United States and South America. All varieties are destined for cultivation in the open field and for fresh market production. We analyzed the genomic diversity using the available SolCAP Tomato Infinium array leading to the detection of 7,591 high-quality SNPs. The maximum likelihood-based phylogenetic analysis better resolved the diversity of the collection, with respect to the Bayesian approach for population structure. The main observed clusters agreed with fruit typologies, thus distinguishing plum and cherry types from beefsteak and globe ones. We did not observe a stratification according to geographical origin except for specific pairs of accessions such as ‘Frassino Locale’ and ‘Vesuviano,’ both from the same district of the Vesuvius area of the Campania region, and ‘San Marzano’ and ‘SMEC,’ two tomato selections from the Agro Nocerino Sarnese area in the Salerno province (Tripodi et al., 2021). The level of heterozygosity did not affect the population structure, confirming previous reports using the same genotyping methodology in cultivated tomato (Schouten et al., 2019). Only by increasing the *K* number of subpopulations, it was possible to observe a degree of admixture for the highly heterozygous accessions. Phenotypic assessment corroborated molecular data showing a partial separation of accessions with different fruit sizes and shapes. The weight, size, and shape of fruits have been the main traits accompanying the domestication and diversification of tomatoes (Paran and van der Knaap, 2007). These results highlight how the genetic diversity of heirlooms has been significantly influenced by breeding and selection focusing on fruit characteristics.

Marker–trait association allowed the detection of interesting candidates underpinning β-carotene and fruit weight. Although the two genic *KIP-1* and *pto-interacting protein 1-like* do not seem to be involved in the variation of carotenoids, we detected a single variant on chromosome 8 matching with *Solyc08g066650.3.1*, which encodes for a *Carotenoid Cleavage Dioxygenase 8* (CCD8). This gene has been reported to play a role in the biosynthesis of apocarotenoids being involved in the conversion of all-*trans*-β-carotene to strigolactones (Kohlen et al., 2012).

For fruit weight, the peak near *Solyc11g069590.2.1* falls at 1 Mbp distance from *SLSUN31* , a *SUN-LIKE* family member reported to be involved in the increase of fruit weight (Tripodi et al., 2021), and at 3 Mbp from *fw11.3*, a major QTL controlling the variation of cell size responsible for the increment of fruit mass. However, this association, although confirmed in both general and mixed linear models, was under the significant threshold when implementing CMLM in GAPIT, thus suggesting further investigations toward the validation of *Solyc11g069590.2.1*.

These results shed light on the potentiality of the diversity enclosed in the heirlooms for gene candidate identification. It must be recognized that the number of individuals used for association analysis is low, although examples are reported with a similar or even a lower sample size (Mazzuccato et al., 2008; Simko et al., 2008; Gitau et al., 2017).

The genotyping strategy used allows for the implementation of genomic data with more individuals, building a broader collection for future genome-wide association mapping studies.

## 5 Conclusion

The increased interest in traditional food staples led to the resurgence of locally grown varieties with distinct nutrition and sensory traits. In this frame, heirlooms are increasingly popular on the market, thanks to their intriguing diversity. In this work, a tomato heirloom collection has been profiled through genomic and metabolic approaches determining qualitative characteristics and genetic diversity and investigating the genetic and environmental factors underlying trait variation. The comprehensive approach used has demonstrated how the set studied provides a broad source of quality-related characteristics. We also highlighted the potentiality of heirloom tomato varieties for gene discovery by generating molecular data through the tomato genotyping array. This strategy allows for the implementation of the collection with new accessions and/or data from previous studies. Overall, the study provides knowledge to determine the importance of heirlooms for their direct consumption or to promote their sustainable use in safeguarding biodiversity and conservation programs and for breeding high-quality products.

## Data availability statement

The original contributions presented in the study are publicly available. These data can be found here: https://figshare.com/articles/dataset/SNP_Data_60_heirlooms_xlsx/21231260.

## Author contributions

PT conceived the project and coordinated the experiments. PT and GF designed the experimental layout. GF and AD’A performed the metabolic characterization. PT performed the genomic analysis. PT and GF collected and curated the data. PT analyzed the data and drafted the manuscript. All authors critically revised the manuscript and approved the submitted version.

## Supplementary material

Supplementary Data Sheet 1 Genotyping matrix using the SolCap array and consisting of 7,591 biallelic SNPs present in over 90% of the 60 tomato heirlooms studied.

Supplementary Figure 1 Distribution of 7,591 SNPs on the 12 tomato chromosomes. The number of SNPs is represented within 1 Mb window size. The horizontal axis shows the chromosome (Chr) length (Mb); each bar represents a chromosome, with Chr 1 at the top and Chr 12 at the bottom. The different colors depict SNP density following the gradient in the legend on the right.

Supplementary Figure 2 Heterozygosity level based on SNP data for the 60 accessions studied.

Supplementary Figure 3 Evaluation of the best grouping number (K) of the Bayesian clustering analysis using the Evanno’s method. (a) Plot of mean likelihood L(K) and variance for 5 independent runs for each value of K, for K = 2–10. (b) Evanno’s plot generated by STRUCTURE HARVESTER for the detection of the true number of clusters (the most likely value of K). c) Table with detailed values for each K. The highest value was at K = 2, indicating that the set studied likely forms 2 sub-populations.

Supplementary Figure 4 Quantile-Quantile (QQ) plots of observed versus expected *P*-values using GLM-naive, GLM-PCA and GLM-Q models for β-carotene (blue line) and fruit weight (green line).
