# Supplementary figures and images for: An integrated genomic and biochemical approach to investigate the potentiality of heirloom tomatoes: Breeding resources for food quality and sustainable agriculture
Source: Front Plant Sci. 2023 Jan 4;13:1031776. doi: 10.3389/fpls.2022.1031776 (PMC9846345; doi:10.3389/fpls.2022.1031776)

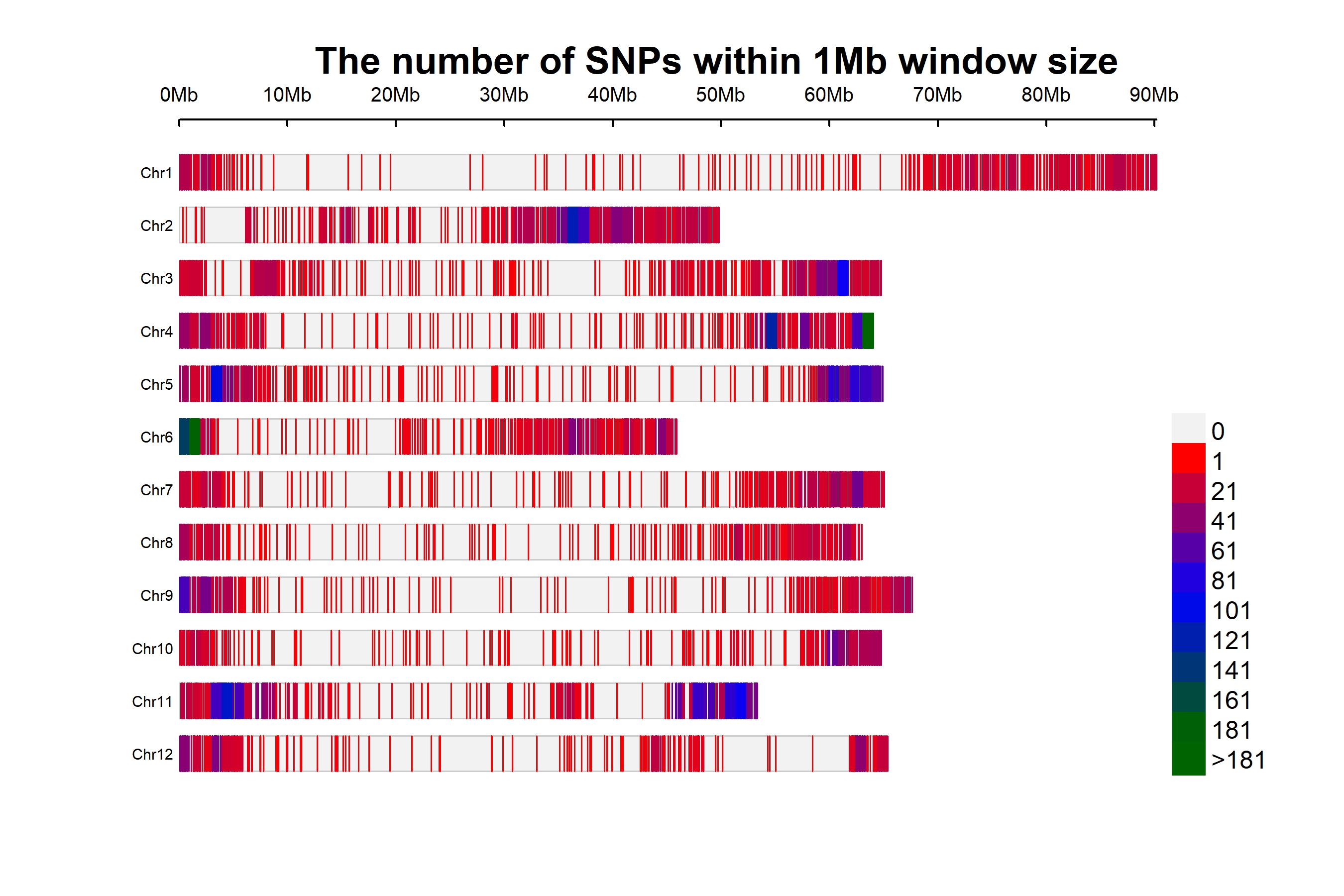

Supplement: Supplementary Figure 1 — Distribution of 7,591 SNPs on the 12 tomato chromosomes. The number of SNPs is represented within 1 Mb window size. The horizontal axis shows the chromosome (Chr) length (Mb); each bar represents a chromosome, with Chr 1 at the top and Chr 12 at the bottom. The different colors depict SNP density following the gradient in the legend on the right. [file Image_1.jpeg]

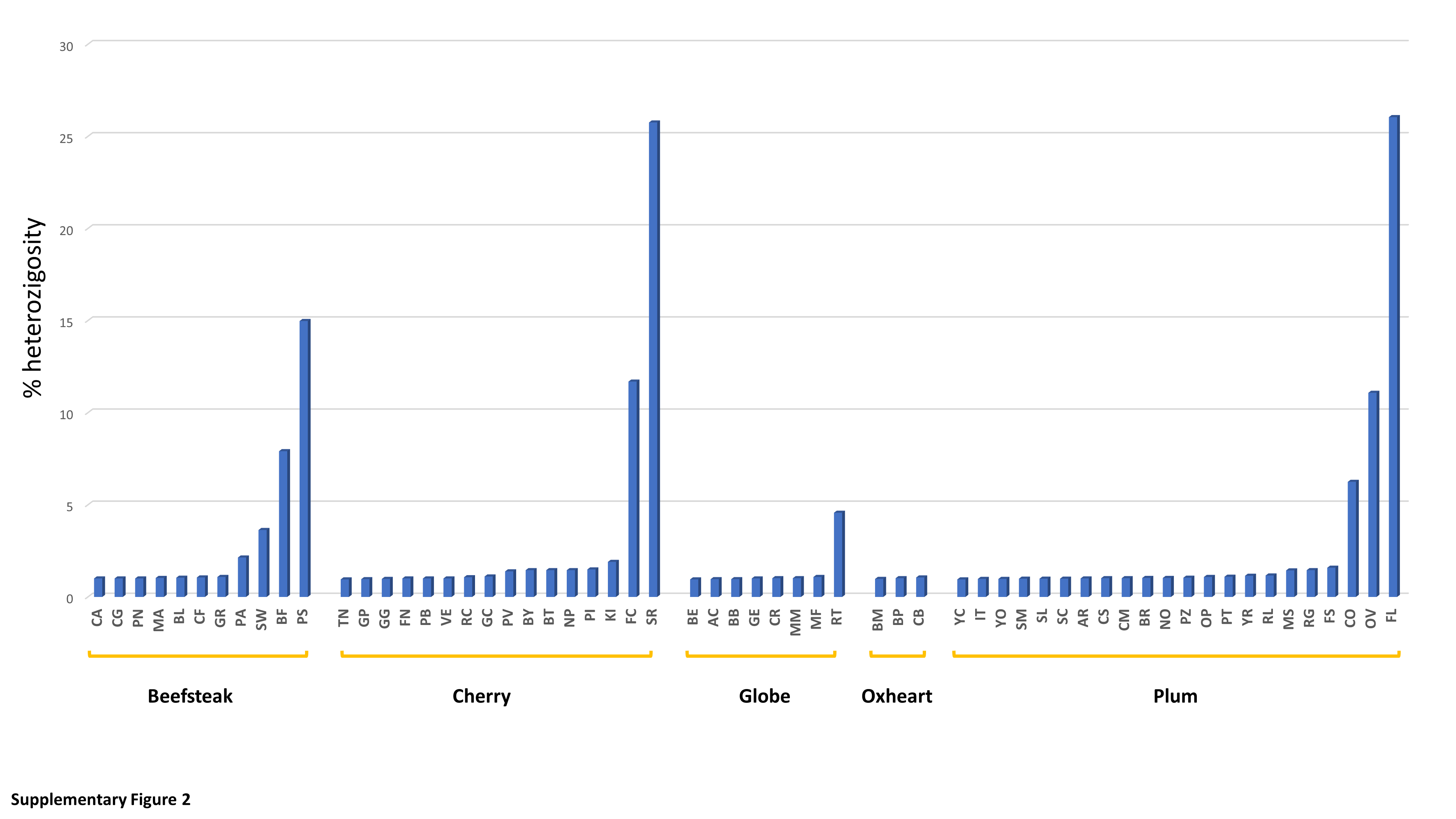

Supplement: Supplementary Figure 2 — Heterozygosity level based on SNP data for the 60 accessions studied. [file Image_2.tif]

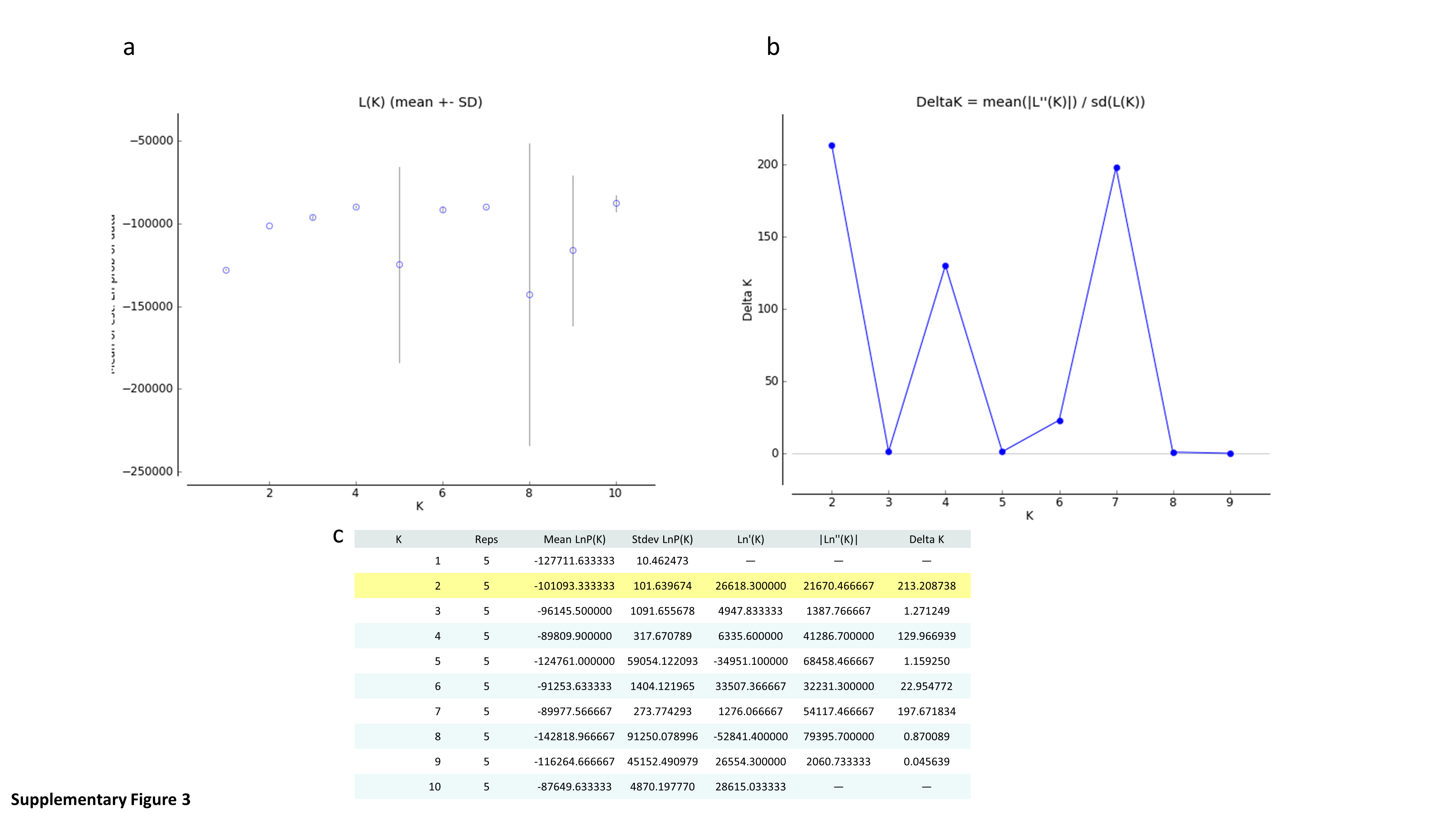

Supplement: Supplementary Figure 3 — Evaluation of the best grouping number (K) of the Bayesian clustering analysis using the Evanno’s method. (a) Plot of mean likelihood L(K) and variance for 5 independent runs for each value of K, for K = 2–10. (b) Evanno’s plot generated by STRUCTURE HARVESTER for the detection of the true number of clusters (the most likely value of K). c) Table with detailed values for each K. The highest value was at K = 2, indicating that the set studied likely forms 2 sub-populations. [file Image_3.tif]

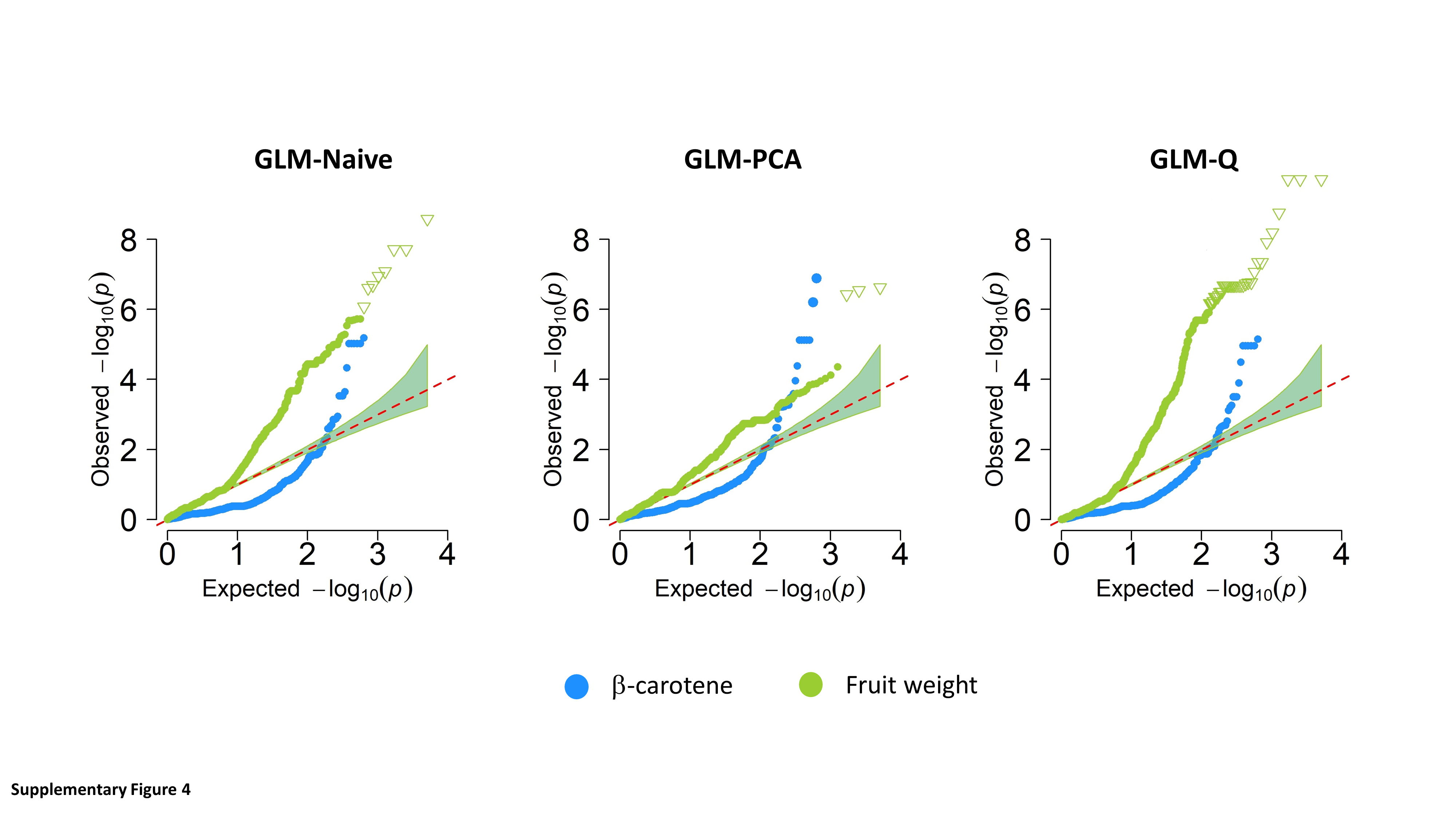

Supplement: Supplementary Figure 4 — Quantile-Quantile (QQ) plots of observed versus expected P-values using GLM-naive, GLM-PCA and GLM-Q models for β-carotene (blue line) and fruit weight (green line). [file Image_4.tif]
